# Correlation between the wide range of tubal pathology discovered by routine hysterosalpingography in a university hospital in Romania and the successful pregnancy rate. A cohort study

**Published:** 2017

**Authors:** R Covali

**Affiliations:** * ”Elena Doamna” Obstetrics and Gynecology University Hospital, Iasi, Romania

**Keywords:** hysterosalpingography, hydrosalpinx, fallopian tube occlusion, fallopian tube patency, contrast agent, successful pregnancy rate

## Abstract

Rationale: Hysterosalpingography is still the main method to begin with when studying the causes of female impossibility to conceive a baby.

Objective: The aim of this study is to correlate and evaluate the wide range of tubal pathology discovered by routine hysterosalpingography in a university hospital in Romania with the successful pregnancy rate.

Methods and Results: A total of 95 consecutive patients explored by routine hysterosalpingography in a university hospital during 2015 and 2016 were included.

Out of 173 fallopian tubes studied, 28.9% were occluded, 13.29% were almost occluded, and only 57.8% were patent.

Of these patients, 11 successful pregnancies occurred in 95 women (11.57%) until September 2017. A number of 7 patients delivered a normal baby in our hospital (7.36%). One patient was admitted at 36 weeks of gestation, and another one at 26 weeks of gestation, for risk of premature delivery. None of these two patients delivered in our hospital. Two patients were admitted for miscarriage at 8 weeks and 5 weeks of pregnancy.

In all the 7 patients who delivered a normal baby, the fallopian tubes were entirely visible (100%), whether they were patent or not.

Discussion: To our knowledge, this is the largest study about hysterosalpingography and the successful pregnancy rate in Romania so far.

Abbreviations: ART= Assisted reproductive technologies

## Introduction

Female fertility is a basic factor for family accomplishment. Lack of children or lack of many children may decrease the self-esteem of the family. New ways of exploring female fertility have been introduced lately. Still, hysterosalpingography is the most widely used method to determine the fallopian tube patency.

Fallopian tube patency is of crucial importance for female fertility. It still is the first and the most important step of exploring uterine and especially tubal status.

This paper aims to evaluate the salpingeal pathology revealed on hysterosalpingography and to correlate it with the pregnancy rate.

## Material and method

**Fig. 1:**
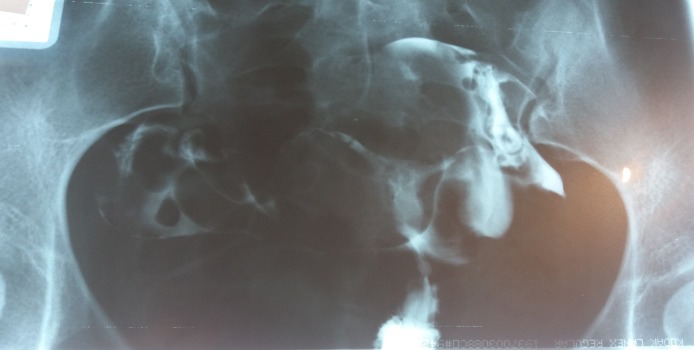
Uterine hypoplasia. Both fallopian tubes are patent.

**Fig. 2:**
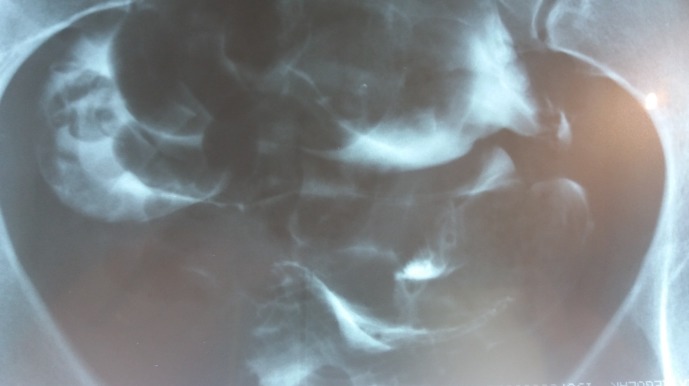
The second film, showing the contrast agent in the whole peritoneal cavity. Evidence that the fallopian tubes are really patent.

**Fig. 3:**
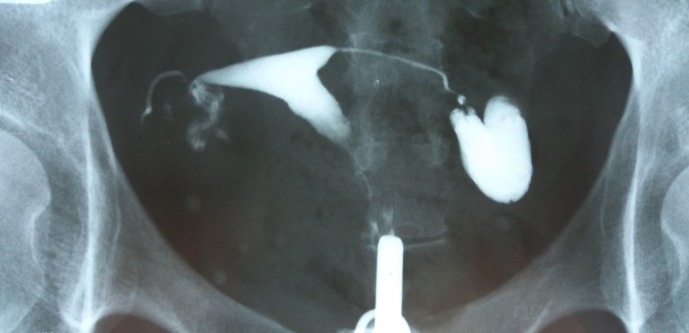
Uterine cavity is normal. The right fallopian tube is entirely visible, looks normal, but a very little amount of contrast agent could pass beyond its end. Left hydrosalpinx.

**Fig. 4:**
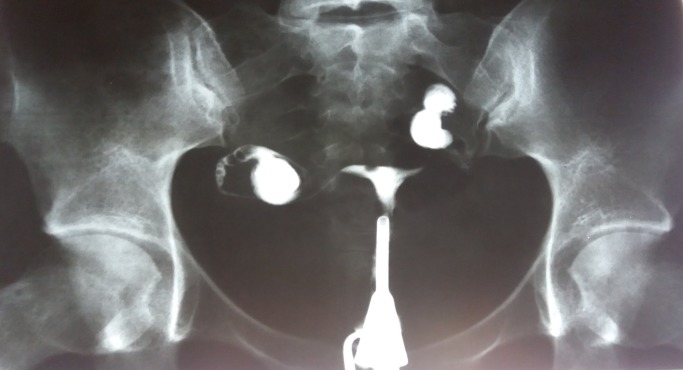
Uterine hypoplasia. Both right and left hydrosalpinx. Spina bifida S1.

A number of 95 consecutive infertility patients, admitted to the university hospital during 2015 and 2016 were studied. Due to the surgical absence of 5 fallopian tubes, and 12 fallopian tubes (n=6 patients) not visible because the uterus could not be filled with contrast agent, only 173 fallopian tubes were studied. During hysterosalpingography, each uterus was filled with up to 10ml water-soluble 370 Omnipaque solution, and one radiograph was performed (**Fig. 1**). In cases in which at least one fallopian tube could not be seen properly, or patency could not be clearly established, a second radiograph, 10 minutes later than the first, was performed. The patient was required to walk or sit during these radiographs. During walking or sitting, the contrast agent descended from the uterus into the vaginal swab. The contrast agent in the fallopian tubes would either flow back in the uterus, and out in the vagina, or flow forward through the tubes to the peritoneal cavity. The second radiograph captured the image of the contrast agent in the peritoneal cavity, and the amount of contrast agent that reached the peritoneal cavity. A large amount of contrast agent in the peritoneal cavity on one side indicated that this particular fallopian tube was patent (**Fig. 2**). Lack of contrast agent in the peritoneal cavity indicated that the tube was not patent. There were cases in which only a small amount of contrast agent could be seen beyond the end of the fallopian tube, and the tube was considered slightly patent (**Fig. 3**). In cases in which the whole salpinx, or only part of it, was visible, but no contrast agent could be detected in the peritoneum, the tube was considered not patent (**Fig. 4**).

Demographic data: The mean age of patients referred for hysterosalpingography was 32.1 years old, with a range between 18 and 43 years old. The mean age of patients who successfully delivered a normal baby was 30.7 years old, with a range between 26 and 34 years old at the referral time. The mean age of patients who got pregnant was 31.09 years old, with a range between 26 and 36 years old. The mean age of patients who suffered a miscarriage was 32.5 years old (29 and 36 years old).

## Results

**Fig. 5:**
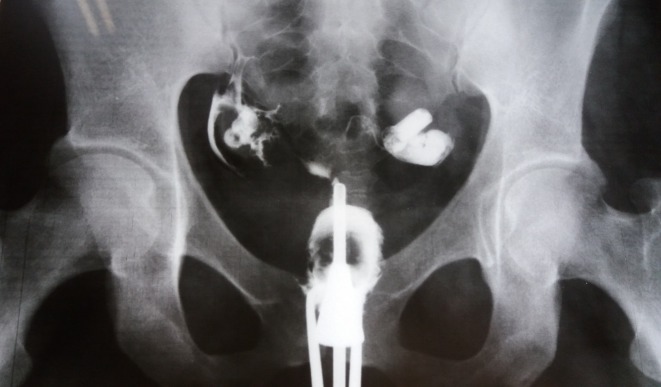
Small uterine cavity. Slightly permissive right fallopian tube. Left hydrosalpinx.

**Fig. 6:**
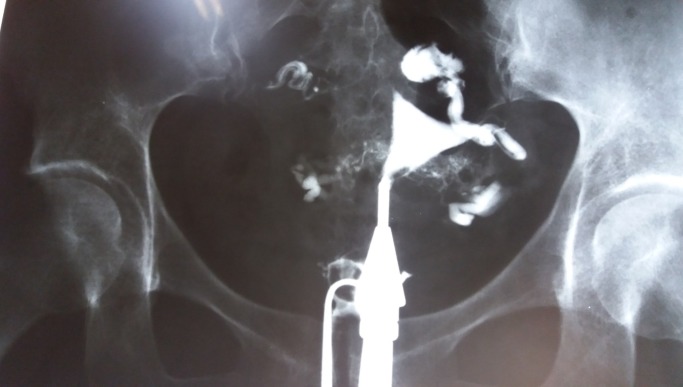
Normal sized uterine cavity. Small isthmical synaechiaea on the left. The right fallopian tube is entirely visible, looks normal, but no contrast agent could pass beyond its end. Left hydrosalpinx . The contrast agent also opacified the periuterine veins, around the lower half of the uterus.

The discovered tubal patency was the following: fallopian tube obstruction in 30.52% (n=29) patients, both salpinges obstructed in 14.73% (n=14) patients, hydrosalpinx in 6.31% (n=6) patients. One of these 6 patients had hydrosalpinx on both sides (1.05%). A total of 24.21% (n=23) fallopian tubes were entirely visible, but a very little amount of contrast agent could pass beyond their end. Both salpinges allowed a very small amount of contrast agent beyond their end in 5.26% (n=5) patients. Hydrosalpinx on one side was associated with a very slightly permissive fallopian tube on the other side in 2.1% (n=2) cases (**Fig. 5**). Hydrosalpinx on one side was associated with an occluded fallopian tube on the other side in 1.05% (n=1) case (**Fig. 6**). Hydrosalpinx on one side was associated with a normal fallopian tube on the other side in 1.05% (n=1) case.

**Fig. 7:**
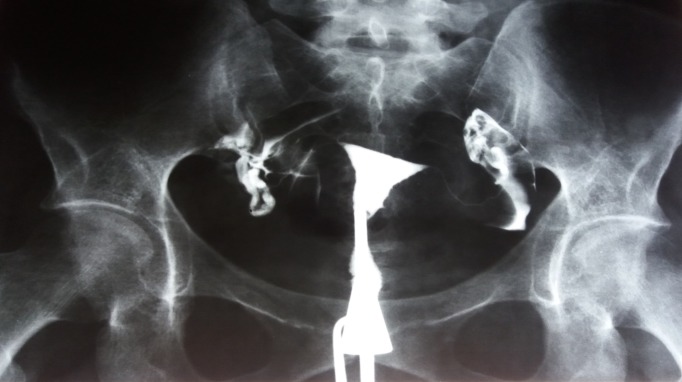
Normal uterine cavity. Right fallopian tube is slightly dilated, but still patent. Normal, patent, left fallopian tube.

**Fig. 8:**
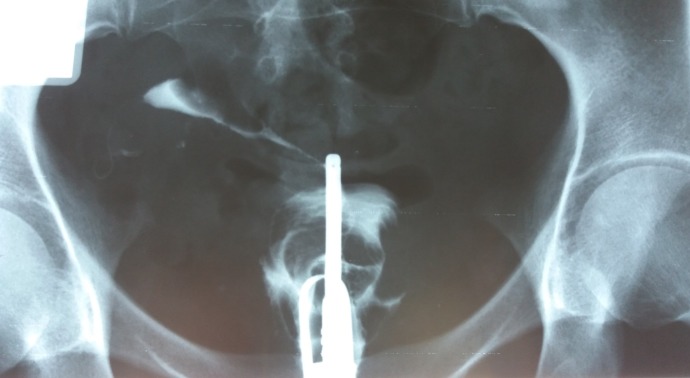
Endometrial polyp. The right fallopian tube is partly visible, but occluded distally. The left fallopian tube is occluded at its uterine insertion, probably by the polyp.

**Fig. 9:**
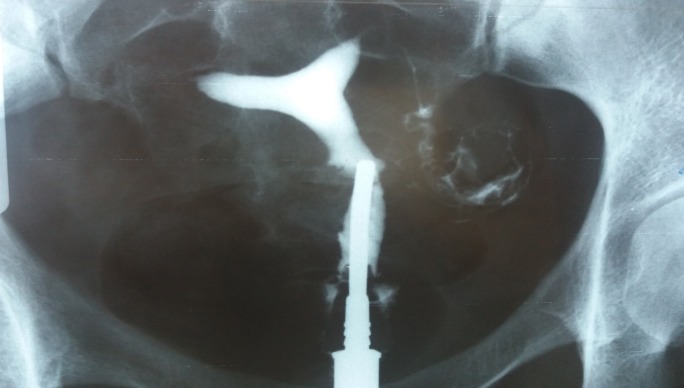
Normal uterine cavity. Occluded right fallopian tube. Normal, patent, left fallopian tube.

**Fig. 10:**
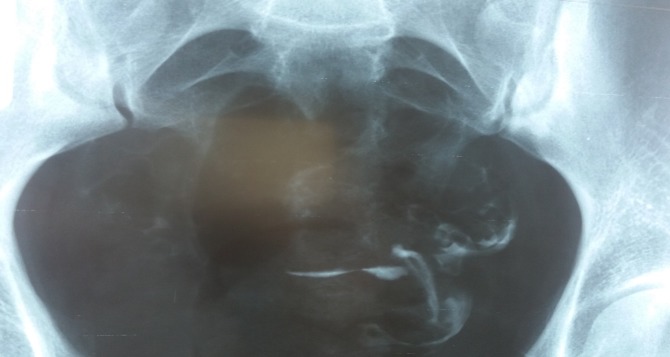
The second film, showing the contrast agent in the left side of the peritoneal cavity. Evidence that the left fallopian tube is really patent. There is also a small amount of contrast agent on the right, most probably originating from the left side of the peritoneal cavity, due to patient movements. This image does not suggest that the right fallopian tube is also patent. The horizontally placed contrast agent in the center of the image represents the contrast agent still left in the uterus.

**Fig. 11:**
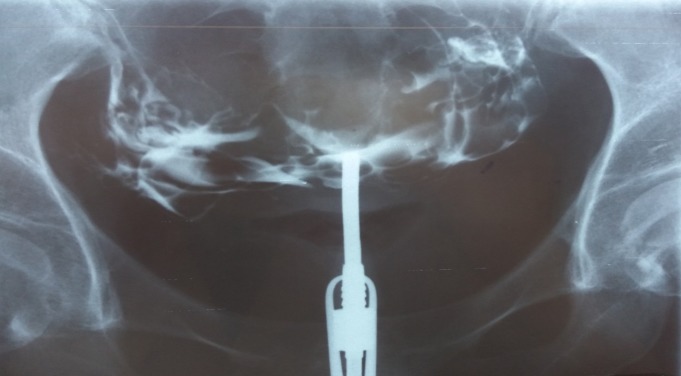
Arcuate uterus. Normal, very patent fallopian tubes. The second film is not necessary.

Out of 173 fallopian tubes studied, 28.9% (n=50) were occluded, 13.29% (n=23) were almost occluded, and only 57.8% (n=100) were patent (Figs. **7**, **8**, **9**, **10**, **11**).

Of these patients, 11 successful pregnancies occurred in 95 women (11.57%) until September 2017. A number of 7 patients delivered a normal baby in our hospital (7.36%). One patient was admitted at 36 weeks of gestation, and another one at 26 weeks of gestation, being considered at increased risk of premature delivery. None of these two patients delivered in our hospital. Two patients were admitted for miscarriage at 8 weeks and 5 weeks of pregnancy.

Of the 7 patients who delivered a normal baby, only 5 patients (62.5%) had both fallopian tubes on hysterosalpingography. The other 2 patients (37.5%) had entirely visible fallopian tubes, but only a small amount of contrast agent could pass beyond their end (both salpinges in one patient, one salpinx in one patient), or no contrast agent could pass beyond its end (one salpinx in one patient). In all the 7 patients who delivered a normal baby, the fallopian tubes were entirely visible (100%), whether they were patent or not.

If both salpinges were visible, but not patent, the chances to get pregnant were 5%. Chances increased if one salpinx was patent (ODDS RATIO 1.35), and they were even higher if both salpinges were patent (ODDS RATIO 2.17).

Of the other 4 patients, the patient admitted at 36 weeks of gestation had a small uterine cavity, not hypoplastic, only slightly smaller than normal. Both salpinges were patent. The patient admitted at 26 weeks of gestation had uterus didelphys. Both salpinges were patent. The 8-week miscarriage patient had an isthmic obstruction that did not allow the entrance of the contrast agent in the uterus, obstruction that must have disturbed the development of the pregnancy. The 5-week miscarriage patient had an arcuate uterus and both salpinges patent.

None of the patients who had fallopian tube obstruction or hydrosalpinx became pregnant, even if the fertility treatment applied in our hospital included restoring patency to the tubes with a wide range of methods, from instillations to laparoscopic neosalpingostomies.

No patient over 40 years old became pregnant. There were 69 patients aged below 35 years, which is considered the best fertility period for women. Out of them, 9 patients (13.04%) got pregnant, and 7 patients (10.14%) delivered a living baby. There were 26 women aged above 35 years, and 2 of them (7.69%) got pregnant. One of them (3.84%) was admitted for surveillance at 36 weeks of pregnancy, but she did not deliver in our hospital. The other one (3.84%) was admitted for miscarriage at 5 weeks of pregnancy.

The mean time elapsed between hysterosalpingography and delivery in the 7 cases above was 15 months (with a range of 10-19 months).

## Discussion including limitations

Hysterosalpingography is the most widely used method for assessing tubal patency. The usual fertility treatments resulted in an overall clinical pregnancy rate of 11.57%, and 13.04% pregnancy rate in women under 35 years old. For this rate to be increased, the intrauterine insemination or assisted reproductive technologies (ART) should be available in our hospital.

Still, even ART treatment does not guarantee pregnancy and live birth [**1**].

Intrauterine insemination results in a cumulative pregnancy rate for live birth per cycle started, over three cycles, of 34.9% [**2**]. In Australia, the overall live birth rate per embryo transfer (ART) was 24.3% in 2013 [**3**]. 

Successful pregnancy rates depend not only on the tubal patency but also on the lifestyle education and the maternal age. Risk factors including smoking, abnormal body mass index or abnormal exercising, alcohol, caffeine consumption, and high levels of stress can decrease even ART clinical pregnancy rate from 46.1% to 19.2% in otherwise unexplained infertility cases [**4**]. The success rates of assisted reproductive technology are decreased among women aged older than 40 years: 23.6% clinical pregnancies and 14.8% live births [**5**]. Regarding specific ART techniques, increasing maternal age benefits more from freeze-only compared to fresh embryo transfer cycles) [**6**].

## Conclusions

Hysterosalpingography remains a good method to determine fallopian tube patency. If associated with ART techniques, successful pregnancy rates can only increase. To our knowledge, this is the largest study about hysterosalpingography and successful pregnancy rate from Romania so far.

## Conflict of interest

The authors declare no conflict of interest.
